# Moderate-intensity treadmill exercise training decreases murine cardiomyocyte cross-sectional area

**DOI:** 10.14814/phy2.12406

**Published:** 2015-05-19

**Authors:** Kathleen Sturgeon, Geetha Muthukumaran, Dennis Ding, Akinyemi Bajulaiye, Victor Ferrari, Joseph R Libonati

**Affiliations:** 1 School of Medicine, University of PennsylvaniaPhiladelphia, Pennsylvania; 2 School of Nursing, University of PennsylvaniaPhiladelphia, Pennsylvania

**Keywords:** Anthracycline, echocardiography, exercise training, heart, murine

## Abstract

The aim of this study was to examine the impact of moderate-intensity treadmill exercise on the structure and function of the murine heart and its associated impact on Akt–AMPK–mTOR signaling. A secondary aim was to test whether the exercise phenotype was altered following a cardiotoxic bolus dose of doxorubicin (DOX). Two-month-old C57Bl/6J female mice remained sedentary (SED,* n* = 12) or were progressively trained with treadmill running for 2 months up to 18 m/min; 60 min/day, 5 days/weeks (EX,* n* = 11) or EX + DOX (15 mg/kg/dose) (EX + DOX,* n* = 6). Following treadmill training, mice underwent graded exercise tolerance testing and echocardiography. Training improved graded exercise tolerance by 68 ± 5% relative to SED, and this effect was not altered with bolus DOX. There were no changes in relative heart size with EX or EX + DOX versus SED. Regional posterior wall thickening was improved in EX and abrogated in EX + DOX. EX had a reduced cardiomyocyte cross-sectional area (CSA) relative to SED, and CSA was further attenuated with DOX. Following EX, AMPK-associated phosphorylation of ULK1(ser317) tended to be lower relative to SED. Akt-associated phosphorylation of TSC2(thr1462) and mTOR(ser2448) were also decreased relative to SED. We observed an increase in AMPK activity with DOX that was not translated to downstream AMPK phosphorylation sites. We conclude that 2 months of moderate treadmill exercise training improves regional cardiac function and exercise capacity, but does not induce relative physiologic hypertrophy in female mice. Differential responses in Akt–AMPK–mTOR signaling may mediate the observed phenotype.

## Introduction

It is well known that aerobic exercise training induces a multitude of cardiovascular adaptations (Wang et al. 2010). Improved myocardial function concomitant with physiologic hypertrophy is commonly observed across species. Studies using rat models have shown an improved functional phenotype and relative cardiac hypertrophy in response to treadmill exercise (Kolwicz et al. 2009), voluntary wheel running (Natali et al. 2001), and swimming (Perrault and Turcotte 1994; Ma et al. 2013). However, less is known regarding the cardiac adaptations to treadmill running in mice, given that swimming is a more frequently utilized exercise modality in this species (Perrino et al. 2011). This is significant because, in rodents, the cardiovascular responses to treadmill exercise have been shown to be quite different from swimming, with differences in hemodynamic adjustments and sympathetic nervous system activation observed between treadmill and swim training (Flaim et al. 1979; Baptista et al. 2008). Moreover, many murine studies have used extreme daily swimming exercise durations that are neither easily quantifiable nor translatable to humans. Given that exercise is often used to compare and contrast signaling mechanisms between physiologic and pathologic hypertrophy and that transgenic mouse models are typically used in this regard, it is imperative to better understand how translationally applicable chronic treadmill exercise training alters the phenotype of the murine heart.

While multiple intracellular signaling pathways are likely involved in exercise-induced cardiac remodeling, swim training in mice has been shown to primarily induce physiologic cardiac hypertrophy through enhanced insulin growth factor-1 (IGF) and protein kinase B (Akt) – mammalian target of rapamycin (mTOR) signaling (McMullen et al. 2007). The mTOR pathway integrates cardiomyocyte energy status with growth factor signaling (McMullen et al. 2007; Zhu et al. 2009; Wang et al. 2012) to regulate a host of processes including cardiomyocyte growth, proliferation, autophagy, and apoptosis. These processes hold complex interactions with feed-forward growth signaling from Akt to the mTOR complex 1 (mTORC1) being antagonized by metabolic stress signaling via AMPK (Arad et al. 2007). Hence, given the central role of Akt–mTOR signaling in swimming-induced cardiac hypertrophy, we investigated the impact of moderate-intensity treadmill exercise on mTOR signaling and its associated impact on the structure and function of the murine heart.

Cardiotoxicity as a consequence of drug treatment acutely alters cardiac structure and/or function. For example, doxorubicin (DOX), is an effective chemotherapeutic agent with broad clinical applications, (Henderson and Frei 1979; Singal and Iliskovic 1998) however, it causes cardiotoxicity and often results in heat failure (Gwinn et al. 2008). Induction of mechanisms associated with pathological cardiac hypertrophy may be counter to mechanisms associated with exercise-induced physiological hypertrophy. Indeed, alterations in mTOR signaling and increased energy stress have been observed in the heart following DOX treatment (Gratia et al. 2012). Despite several studies showing a prophylactic cardioprotective effect of exercise prior to DOX administration (Scott et al. 2011), little is known about whether DOX alters the Akt–AMPK–mTOR axis in the trained heart (Ashour et al. 2012; Singla 2014). Thus, here we utilize DOX as a tool to perturb hypothesized cardiac adaptations to exercise training. The adaptive, or “trained”, phenotype was hypothesized to have marked differences in cardiac structure, function, and molecular signaling with comparison to cardiac function and molecular signaling in sedentary animals and in exercise trained animals exposed to an acute pathological stressor.

## Methods

### Animals and experimental protocol

Six-to-eight-week-old female C57Bl/6J mice (Jackson Laboratories) were randomly divided into the following cohorts: sedentary (SED, *n* = 12), exercise (EX, *n* = 11), and exercise + doxorubicin (EX + DOX, *n* = 6). A 5 day/week progressive exercise training program was utilized such that time and intensity incrementally increased from 30 min at 12 m/min in week 1 to 60 min at 18 m/min by week 5 and maintained through week 8 of training. Forty-eight hours after the last exercise session, EX + DOX animals received a bolus IP injection of 100 *μ*L PBS containing 15 mg/kg doxorubicin HCL (Sigma Aldrich, St. Louis, MO). SED and EX animals received a bolus IP injection of 100 *μ*L PBS. A subset of SED, EX, and EX + DOX animals completed baseline and follow-up maximal exercise testing. Maximal exercise tests consisted of 3 min stages starting at 10 m/min and increasing 3 m/min at 0% incline until exhaustion (Mille-Hamard et al. 2012). All experiments were approved by the Institutional Animal Care and Use Committee of The University of Pennsylvania, and all animals received humane care in compliance with NIH standards.

### Echocardiography

Seven days following the last exercise training session, and 5 days following DOX administration, a subset of mice were sedated using 3% isoflurane, and a Vevo 770 (VisualSonics, Toronto, Canada) ultrasound machine with a 30 MHz probe was used for cardiac imaging. As previously described (Sturgeon et al. 2014), a two-dimensional mode with short-axis views at the level of the papillary muscles was used to measure diastolic and systolic left ventricle anterior wall (LVAW) thickness, LV internal diameter (LVID), and LV posterior wall (LVPW) thickness (SED: *n* = 6; EX: *n* = 5; EX + DOX: *n* = 6). Four-chamber apical views were obtained for Doppler tissue imaging of early diastolic peak velocity (E wave) and late diastolic peak velocity (A wave). LV volumes, fractional shortening (FS), and LV mass were then calculated from cardiac measurements (LV Vol = ((7.0/(2.4 + LVID))*LVID^3^, FS = 100*((LVID;d − LVID;s)/LVID;d), LV mass = 1.053*((LVID;d+LVPW;d  +  LVAW;d)^3^-LVID;d).

### Histological analysis

Following an overdose of isoflurane, hearts were extracted via thoracotomy, sectioned such that a basal sample was fixed in 4% paraformaldehyde and an apical sample was frozen in liquid nitrogen (SED: *n* = 6; EX: *n* = 5; EX + DOX: *n* = 6). Fixed sections were embedded in paraffin and stained with hematoxylin and eosin (H&E) and Masson's trichrome. Photographs of five cross sections for each animal were generated with a mounted digital camera (DS-Qi1Mc; Nikon, Tokyo, Japan) and light microscope (BX-FLA; Olympus, Shinjuku, Japan). To determine cardiomyocyte cross-sectional areas (CSA), H&E slides were analyzed by outlining round to cuboidal-shaped nucleated myocytes. Percent fibrosis was calculated as amount of collagen per myocardial cross section.

### Western blots

Western blotting was performed on snap-frozen tissue samples (SED: *n* = 3; EX: *n* = 3; EX + DOX: *n* = 3). Tissue lysates were prepared by homogenization and the protein concentration was assessed as previously described (Kolwicz et al. 2009). Equal amounts of protein (50 *μ*g) were separated by SDS-PAGE and transferred to nitrocellulose membrane (BioRad, Hercules, CA). Primary antibodies for: phosphorylated (p)-AMPK at threonine(thr)172, AMPK, p-Akt at serine (ser) 473, Akt, p-TSC2(ser1387), p-TSC2(thr1462), TSC2, p-mTOR(ser2481), p-mTOR(ser2448), mTOR, p-Raptor(ser792), Raptor, p-p70S6K(thr387), p70S6K, p-ULK1(ser317), p-ULK1(ser757),and ULK1 (Cell Signaling, Danvers, MA) were used. Signals were visualized by enhanced chemiluminescence, digitized, and quantified with Image J software (NIH, Bethesda, MD).

### Statistical analysis

All values are reported as mean ± SEM. Differences in physiological, histological, and western blots were analyzed by one-way ANOVA followed by Scheffe's post hoc test. All statistical testing was carried out by STATA 12.1 (College Station, TX). An alpha level of <0.05 was deemed significant. Our power ranged from 87% to 100% for all significant findings.

## Results

### Functional capacity and whole heart structure and function

**Figure 1:**
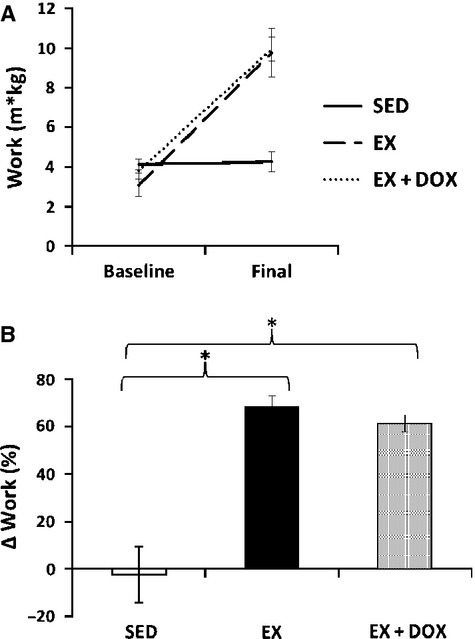
Maximal exercise testing. C57BL/6J female mice were exercise trained over 2 months prior to investigating cardiac function and cellular signaling. Maximal treadmill exercise tests (speed starting at 10 m/min with 3 m/min increases every 3 min until exhaustion) were performed at baseline and after training (final) in a subset of animals: sedentary (SED, *n* = 6), exercise trained (EX, *n* = 5), and EX + doxorubicin (DOX, *n* = 6) treated animals (15 mg/kg bolus following exercise training program). A significant increase in work was seen at final testing in EX and EX + DOX groups (A), and the percent change in work from baseline levels was also significantly (*P <* *0.001) higher in EX and EX + DOX animals relative to SED animals (B).

Exercise training resulted in a significant increase in work capacity that was unchanged in the presence of DOX treatment (Fig.1). This improvement in work capacity was independent of exercise-induced cardiac hypertrophy, as body weight, absolute heart weight, heart weight-to-body weight ratio, tibial length, and heart weight-to-tibia length ratio were not different between groups (Table1). On echocardiography there were significant differences in regional posterior wall function, but little change between groups in LV mass or volumes, ejection fraction, or E/A ratio (Table2). EX animals had greater percent posterior wall thickening compared to SED animals (*P* < 0.05), and this exercise effect was mitigated with DOX (*P* < 0.01).

**Table 1:** Physical characteristics

Physical characteristics	Sedentary (*n* = 12)	Exercise (*n* = 11)	Exercise + DOX (*n* = 6)
Posttraining BW (g)	21.5 ± 0.38	20.6 ± 0.32	20.8 ± 0.66
BW at sacrifice (g)	20.6 ± 0.36	21.6 ± 0.76	21.1 ± 0.49
Heart weight (mg)	94.7 ± 3.02	94.9 ± 4.21	89.2 ± 3.50
Heart weight/BW (mg/g)	4.4 ± 1.65	4.6 ± 0.21	4.2 ± 0.17
Tibia length (mm)	18.5 ± 0.21	17.9 ± 0.22	18.6 ± 0.21
Heart weight/TL (mg/mm)	5.1 ± 0.16	5.3 ± 0.27	4.8 ± 0.21

No differences in physical characteristics were observed between sedentary, exercise trained, and exercise-trained mice given a bolus dose of doxorubicin (DOX). Body weight (BW), tibia length (TL).

**Table 2:** Echocardiographic measures of cardiac function

Variable	Sedentary (*n* = 6)	Exercise (*n* = 5)	Exercise + DOX (*n* = 6)	*P*-value
LVID; d (mm)	3.7 ± 0.10	3.5 ± 0.05	3.7 ± 0.14	0.65
LVID; s (mm)	2.6 ± 0.14	2.5 ± 0.14	2.5 ± 0.14	0.77
LVPW; d (mm)	0.7 ± 0.03	0.8 ± 0.09	0.8 ± 0.05	0.56
LVPW; s (mm)	1.0 ± 0.05	1.3 ± 0.18	1.0 ± 0.07	0.09
LVAW; d (mm)	0.8 ± 0.04	0.7 ± 0.06	0.8 ± 0.05	0.18
LVAW; s (mm)	1.1 ± 0.08	1.1 ± 0.07	1.1 ± 0.06	0.96
LV volume; d (*μ*L)	57.2 ± 3.65	52.4 ± 1.64	57.8 ± 5.42	0.62
LV volume; s (*μ*L)	26.4 ± 3.85	23.4 ± 3.25	23.4 ± 2.96	0.76
Stroke Volume (*μ*L)	30.7 ± 2.49	29.0 ± 2.85	34.4 ± 3.54	0.46
Ejection fraction (%)	54.4 ± 4.19	55.6 ± 5.88	59.8 ± 3.57	0.67
Heart rate (bpm)	391.8 ± 18.27	396.6 ± 24.08	415.8 ± 17.40	0.65
Temperature (°C)	36.3 ± 0.76	37.2 ± 0.31	36.9 ± 0.48	0.60
Fraction shortening (%)	27.9 ± 2.54	28.8 ± 3.73	31.4 ± 2.37	0.65
Posterior wall thickening (%)	29.3 ± 2.41	37.4 ± 2.44	17.7 ± 4.60^a^	0.004
LV mass (mg)	79.9 ± 5.79	72.3 ± 6.12	84.4 ± 7.60	0.46
MV E (mm/sec)	576.5 ± 42.73	654.6 ± 63.82	534.4 ± 61.40	0.35
MV A (mm/sec)	365.2 ± 23.35	471.2 ± 63.15	413.4 ± 40.89	0.35
E/A	1.6 ± 0.18	1.4 ± 0.05	1.3 ± 0.08	0.16

Animals that exercised for 2 months did not change cardiac volume, mass, or diastolic (d) function, but did demonstrate significantly enhanced posterior wall thickening during systole (s). Left ventricle (LV) internal diameter (LVID), LV posterior wall (LVPW), LV anterior wall (LVAW), mitral valve early wave peak (MV E), mitral valve atrial wave peak (MV A)

a *P* < 0.01 between exercise and exercise + doxorubicin (DOX) groups.

### Cardiomyocyte CSA and LV fibrosis

**Figure 2:**
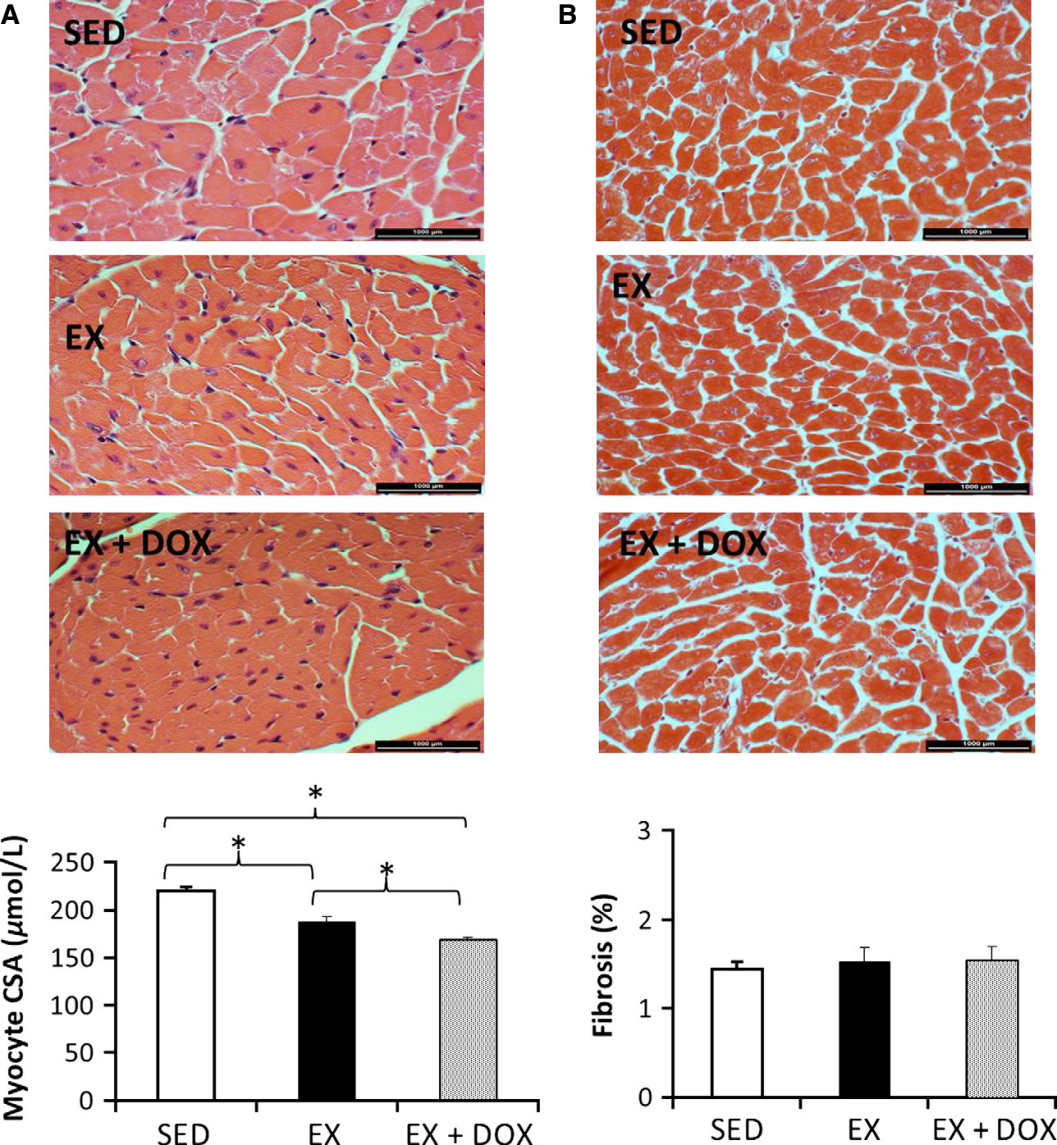
Histological analysis of cardiomyocyte cross sectional area and cardiac fibrosis. Representative images of cardiomyocyte cross sectional area and quantification (A); and representative images of cardiac fibrosis and quantification (B), demonstrated that cardiomyocyte cross sectional area was significantly decreased with exercise (EX, *n* = 5) and doxorubicin (DOX, *n* = 6) exacerbated this effect (*P < 0.001) compared to sedentary (SED, *n* = 6) animals. However, cardiac fibrosis was not different between groups.

The CSA of cardiomyocytes was significantly smaller in hearts isolated from EX mice as compared to SED mice, whereas the addition of DOX treatment to EX decreased cardiomyocyte CSA even further (Fig.2A). We did not observe any differences in cardiac fibrosis between groups (Fig.2B).

### mTOR signaling proteins

**Figure 3:**
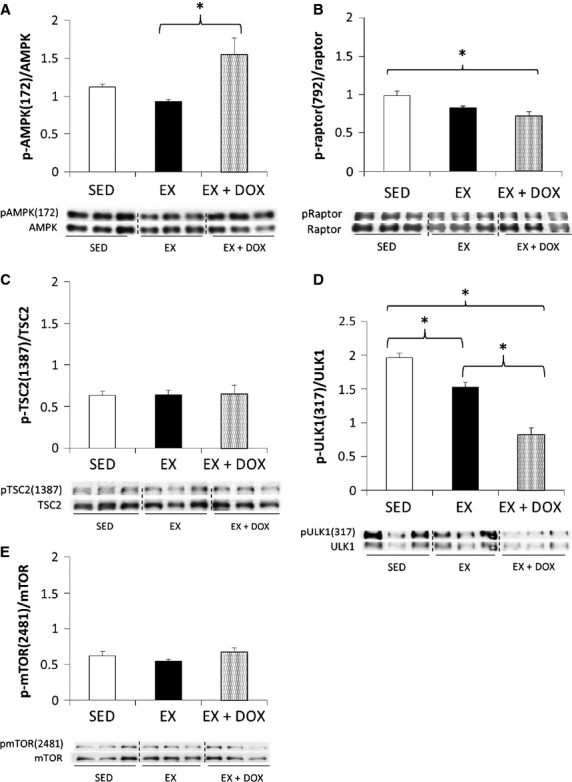
Western blotting analysis of cardiac AMPK-mTOR-autophagy signaling. Sedentary (SED, *n* = 3), exercise trained (EX, *n* = 3), and EX mice given a bolus injection of doxorubicin (DOX, *n* = 3) revealed hypo-phosphorylation patterns of AMPK phosphorylation sites following EX training. Additionally, increased AMPK activity with DOX did not result in downstream increased phosphorylation patterns of AMPK phosphorylation sites following DOX treatment. *P <* *0.05.

The mTOR pathway is a critical regulator of cell growth and its activity is known to be altered during cardiac hypertrophy and heart failure. Both AMPK and Akt are upstream of mTOR, thus we examined the activity of these kinases during EX and in combination with DOX to determine whether their activity affected cardiomyoctye CSA. There was a trend for decreased AMPK activity in EX animals compared to SED animals (t-test between SED and EX *P* = 0.009; ANOVA *P* = 0.14). The addition of bolus DOX increased AMPK activity in EX + DOX animals compared to EX alone (*P* < 0.05, Fig.3A). Activity at the AMPK phosphorylation site on Raptor, serine 792, was diminished following exercise training (Fig.3B), and in EX + DOX animals, activity at Raptor(ser792) was significantly less phosphorylated (*P* < 0.05). Activity at the AMPK phosphorylation site on ULK1, serine 317, was significantly diminished in both EX and EX + DOX animals (*P* < 0.05; Fig.3D). Altho-ugh exercise affected these downstream AMPK targets through attenuating phosphorylation, the DOX-induced increase in AMPK activity did not affect downstream targets. Furthermore, we did not observe any changes in activity of the AMPK phosphorylation site on TSC2 (serine 1387) (Fig.3C) with EX or EX + DOX. The auto-phosphorylation site of mTOR (serine 2481) was not altered between groups (Fig.3E).

**Figure 4:**
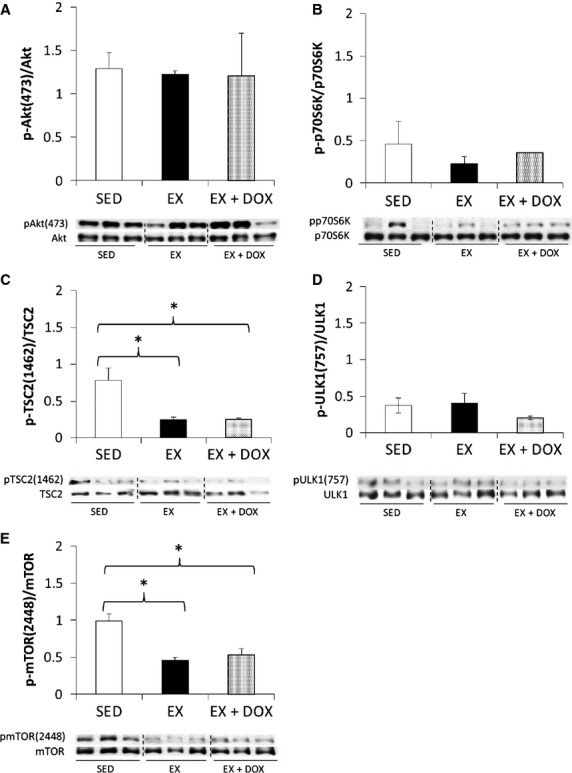
Western blotting analysis of cardiac Akt-mTOR-autophagy signaling. Sedentary (SED, *n* = 3), exercise trained (EX, *n* = 3), and EX mice given a bolus injection of doxorubicin (DOX, *n* = 3) mice revealed while Akt signaling was not altered, there was significant hypo-phosphorylation at TSC2 and mTOR phosphorylation sites specific for Akt. DOX did not cause any significant differences in Akt mediated signaling. *P <* *0.05.

As mTOR is impacted by both AMPK and Akt, we also determined signaling shifts in Akt. We did not observe any changes in Akt activity for EX or EX + DOX animals as indicated by the serine 473 phosphorylation site (Fig.4A). Downstream of Akt, p70S6K was not altered, nor was ULK1 (serine 757) (Fig.4B and D, respectively). Independent of alterations in Akt activity, we observed less phosphorylation at the Akt phosphorylation site on TSC2 (threonine 1462) and mTOR (serine 2448) for both EX and EX + DOX animals compared to SED animals (Fig.4C and E, respectively).

## Discussion

The major findings of this study are that while treadmill training resulted in a significant increase in work capacity, bolus DOX administration did not affect this parameter. The training-induced improvement in work capacity was largely independent of resting phenotypical changes in the heart. Structurally, heart mass remained unchanged with treadmill training and we even observed smaller cardiomyocyte cross-sectional surface areas in trained hearts that were further attenuated with bolus DOX posttraining. While training improved posterior wall regional function relative to sedentary hearts, we did not observe whole heart functional alterations on echocardiography. Interestingly, bolus DOX administration attenuated the improvement of posterior wall thickening that occurred with training. Neither Akt nor mTOR(ser2481) were significantly altered with treadmill training, and a reduced phosphorylation of the downstream effector protein ULK1 was observed, suggestive of diminished autophagy activity. Even though AMPK activity was increased with DOX, consistent with added energy stress on the heart, the training-induced reduction in ULK1 persisted. Training also attenuated TSC2(thr1462) and mTOR(ser2448), neither of which was effected by bolus DOX. Collectively our results suggest that moderate-intensity treadmill training has little effect on physical characteristics such as relative hypertrophy, mTOR signaling, or resting function in the female mouse heart. Our results also suggest that while bolus DOX does alter elements of the cardiac mTOR signaling pathway following exercise training, it does not mitigate the accrued improvements in exercise capacity.

High-intensity training models have successfully been utilized to alter cardiac phenotype in rodents (Kemi et al. 2005; Iemitsu et al. 2006). In our study, we utilized a moderate-intensity progressive training program that reached an estimated intensity of approximately 65–70% VO_2max_ (Fernando et al. 1993). This relates to approximately a 11–13 (fairly light to somewhat hard, or, brisk walking/light jogging) rating on the Borg scale of perceived exertion for humans and is translationally applicable (Borg 1982; Eston et al. 1987; Eston and Williams 1988). Several treadmill-based studies in mice have also used translationally applicable exercise loads (Desai et al. 1997; De Angelis et al. 2004; Mehl et al. 2005; Baltgalvis et al. 2008). While some studies have demonstrated modest cardiac hypertrophy (12.6% increases in mice relative to 30% increases in rats), others have reported limited or no effects on cardiac structure (Fewell et al. 1997; Rosa et al. 2005; Bellafiore et al. 2007; Han 2013). Specifically, Bellafiore et al., reported increases in LV dimensions with training, but did not see an increase in cardiomyocyte size or number (Bellafiore et al. 2007). Additionally, Fewell et al. and Han did not observe hypertrophic responses to moderate treadmill training in mice (Fewell et al. 1997; Han 2013). Thus, our findings of no changes in absolute or relative heart sizes with treadmill exercise are in accordance with previous work using translationally applicable exercise loads. Additionally, we observed marginal exercise-induced improvements in cardiac function, that is, increased posterior wall thickening. Regional changes in posterior wall thickening are indicative of improved regional contractility and enhanced cardiac contractility is a hallmark adaptation to exercise (Kaurstad et al. 2012). While cardiac improvements are undoubtedly related to exercise capacity, in our study, DOX did not result in a lesser work capacity even though it greatly attenuated posterior wall contractility. This suggests the involvement of other cardiac and peripheral mechanisms in establishing work capacity.

Treadmill exercise training studies using rodents do not consistently report significant increases in cardiomyocyte size (Kemi et al. 2005), and myocardial structural shifts appear to be related to both exercise mode and intensity. Kemi et al. report improvements in cardiomyocyte length in rats, but not cardiomyocyte CSA with moderate exercise (Kemi et al. 2005). Furthermore, Bellafiore et al. reported no changes in murine cardiomyocyte size with 15, 30, or 45 days of progressive moderate treadmill training (Bellafiore et al. 2007). However, we are the first to report a significant decrease in cardiomyocyte CSA following clinically applicable moderate levels of treadmill running. This result was surprising and contrary to our initial hypotheses. It does not, however, negate the possibility of exercise-induced cardiomyocyte eccentric hypertrophy, or increased cardiomyocyte length, a variable not determined in our study.

While multiple intracellular signaling pathways are likely involved in exercise-induced cardiac remodeling, swim training in mice has been shown to primarily induce physiologic cardiac hypertrophy through the IGF–Akt–mTOR pathway (McMullen et al. 2007). This pathway is crucial in energy sensing, growth factor signaling, and the establishment of cardiomyocyte number and size. In studies that have utilized swimming or high-intensity treadmill training, activation of Akt(ser473), TSC2(thr1462), and mTOR(ser2448) have been observed (Kemi et al. 2008; Ma et al. 2013). However, much less is known about the activity of accessory proteins such as Raptor or the activity of AMPK-specific phosphorylation sites within the mTOR signaling pathway with translationally applicable levels of treadmill exercise. Hence, given the primary central role of Akt–mTOR signaling in swimming-induced cardiac hypertrophy, we explored the impact of moderate-intensity treadmill exercise on mTOR signaling and then perturbed the pathway with bolus DOX administration.

The cardiac mTOR signaling data in our study supports the lack of progrowth signaling with treadmill exercise. Following exercise training, we did not observe Akt-induced mTOR progrowth signaling as Akt and p70S6K activity was not different between groups. The attenuated phosphorylation of mTOR(ser2448) and TSC2(thr1462) with EX and EX + DOX relative to SED was further indicative of diminished progrowth signaling. TSC2 is inactivated by Akt-dependent phosphorylation, which allows Rheb-GTP to accumulate and bind directly to mTOR within mTORC1. Thus, with lower phosphorylation, TSC2 is active and Rheb-GDP is dominant (Huang and Manning 2008). This classical pathway for cell growth was not affected by EX or doxorubicin-induced changes in AMPK, as indicated by the AMPK-specific phosphorylation site on TSC2 and the autophosphorylation site on mTOR, serine 2481.

In an alternative pathway of regulation, Raptor, an accessory protein necessary for mTORC1 and subsequent growth was less phosphorylated with training. This suggests that Raptor is enabled to complex with mTOR to form mTORC1 (Gwinn et al. 2008). This potential priming for a progrowth cascade is paired with observations of anti-autophagy signaling. Under conditions of starvation and enhanced AMP levels, AMPK promotes autophagy by directly phosphorylating ULK at serine 317 (Kim et al. 2011). However, we observed decreased activity of ULK1(ser317) with exercise. Additionally, while DOX increased AMPK activity, the lower phosphorylation patterns on Raptor and ULK1 were still maintained. Hence, while our data suggest less autophagy with treadmill exercise, these effects were counterbalanced by antigrowth signaling in mTOR.

Collectively, our data demonstrate the lack of exercise-induced relative cardiac hypertrophy in a murine model using translatable exercise training intensity, duration, and modality. Not only were there no differences in cardiac dimensions and traditional indicators of cardiac function, but we observed a significant decrease in murine cardiomyocyte CSA with exercise that was enhanced with acute DOX exposure. We observed reduced activity of autophagy signaling with exercise but these shifts were counterbalanced by less TSC2 and mTOR activity. We conclude that more research is needed to investigate cardiac adaptations to exercise mode and intensity. Given that exercise is often used to compare and contrast signaling mechanisms between physiologic and pathologic hypertrophy and that transgenic mouse models are typically used in this regard, it is imperative to better understand how translationally applicable chronic treadmill exercise training alters the phenotype of the murine heart.
